# Supplementary material for: Using iPALM to determine protein organisation in cardiac muscle Z-discs
Source: PLoS Comput Biol. 2026 Sep 28;22(9):e1014309. doi: 10.1371/journal.pcbi.1014309 (PMC13638043; doi:10.1371/journal.pcbi.1014309)
Supplement: Supplementary file 1 [file pcbi.1014309.s005.docx]

**Table S1. Hyperparameter sweep.** Parameter values used in the exhaustive sweep over possible experimental RPDs and models for each protein and direction combination. σ_loc_: maximum estimated localisation precision filter. N_locszdisc_: minimum number of localisations in a Z-disc for inclusion of the Z-disc. L_fit_: Distance over which the model was fitted. N_peaks_: number of characteristic distances included in the model RPD. d_type_: Relationship between characteristic distances in the model (**Methods**). Repeats: Inclusion of a term for repeated localisations of a single molecule.

|  |  | **Experimental RPD** | | | **Model** | | | |
| --- | --- | --- | --- | --- | --- | --- | --- | --- |
| **Protein** | **Direction** | **σ_loc_ (nm)** | **N_locszdisc_** | **L_fit_ (nm)** | **Background** | **N_peaks_** | **d_type_** | **Repeats** |
| ɑ-actinin-2 | Axial | 5, 6, 7 | 1, 5, 10 | 105, 115, 125 | None, Flat, Linear_1D_, Linear_flat_ | 0, 1, 4, 5, 6 | int | False,  True |
| ZASP | Axial | 9, 10 | 1 | 110, 115, 120 | None, Flat, Linear_1D_, Linear_flat_ | 0, 1, 4, 5, 6 | int | False,  True |
| Z1Z2 | Axial | 7, 8, 9 | 1, 5, 10 | 100, 110,120 | None, Flat, Linear_1D_, Linear_flat_ | 0, 1, 4, 5, 6 | int | False,  True |
| ɑ-actinin-2 | Transverse | 5, 6, 7 | 1, 5, 10 | 52, 57, 62 | None, Flat, Linear_2D_, Linear_2D,int=0_, Quad_2D_, Cubic_2D_ | 0, 2, 3 | int,  sq,  ind | False,  True |
| ZASP | Transverse | 9, 10 | 1 | 42, 47, 52 | None, Flat, Linear_2D_, Linear_2D,int=0_, Quad_2D_, Cubic_2D_ | 0, 2, 3 | int,  sq,  ind | False,  True |
| Z1Z2 | Transverse | 7, 8, 9 | 1, 5, 10 | 50, 55, 60 | None, Flat, Linear_2D_, Linear_2D,int=0_, Quad_2D_, Cubic_2D_ | 0, 2, 3 | int,  sq,  ind | False,  True |
