# Supplementary material for: Using iPALM to determine protein organisation in cardiac muscle Z-discs
Source: PLoS Comput Biol. 2026 Sep 28;22(9):e1014309. doi: 10.1371/journal.pcbi.1014309 (PMC13638043; doi:10.1371/journal.pcbi.1014309)
Supplement: Supplementary file 2 [file pcbi.1014309.s006.docx]

**Table S2 Pre-processing parameters for final experimental RPD.** Filters used to obtain the experimental RPD for final model selection for each protein and direction.

| **Protein** | **Direction** | **σ_loc_ / nm** | **N_locszdisc_** | **L_fit_ / nm** | **N_distances_** |
| --- | --- | --- | --- | --- | --- |
| ACTN2 | Axial | 6 | 1 | 125 | 457 |
| Z1Z2 | Axial | 8 | 1 | 120 | 406 |
| ZASP | Axial | 9 | 1 | 120 | 69 |
| ACTN2 | Transverse | 6 | 1 | 62 | 462 |
| Z1Z2 | Transverse | 7 | 1 | 60 | 134 |
| ZASP | Transverse | 9 | 1 | 52 | 69 |
