# Supplementary material for: Using iPALM to determine protein organisation in cardiac muscle Z-discs
Source: PLoS Comput Biol. 2026 Sep 28;22(9):e1014309. doi: 10.1371/journal.pcbi.1014309 (PMC13638043; doi:10.1371/journal.pcbi.1014309)
Supplement: Supplementary file 3 [file pcbi.1014309.s007.docx]

**S3 Table.** **Most likely models and parameter values for ɑ-actinin-2 arrangement.**

| **Axial** | **Background:** linear_flat_ | Slope (a.u. nm^-1^) | -0.10 ± 0.00 |
| --- | --- | --- | --- |
|  |  | Offset (a.u.) | 5.51 ± 0.17 |
|  | **Characteristic distances:** linear repeat, 6 peaks | Repeat distance (nm) | 16.25 ± 0.09 |
|  |  | Amplitude 1 (a.u.) | 65.89 ± 3.42 |
|  |  | Amplitude 2 (a.u.) | 63.88 ± 0.91 |
|  |  | Amplitude 3 (a.u.) | 71.77 ± 1.94 |
|  |  | Amplitude 4 (a.u.) | 60.39 ± 2.29 |
|  |  | Amplitude 5 (a.u.) | 13.50 ± 1.96 |
|  |  | Amplitude 6 (a.u.) | 33.12 ± 1.55 |
|  |  | Broadening (nm) | 11.47 ± 0.33 |
|  | **Repeated localisations** | Not included | |
| **Transverse** | **Background:** linear_2D,int=0_ | Slope (a.u. nm^-1^) | 0.06 ± 0.00 |
|  | **Characteristic distances:** independent distances, 3 peaks | Distance 1 (nm) | 10.32 ± 0.03 |
|  |  | Amplitude 1 (a.u.) | 5.75 ± 0.07 |
|  |  | Distance 2 (nm) | 30.00 ± 0.06 |
|  |  | Amplitude 2 (a.u.) | 14.72 ± 0.23 |
|  |  | Distance 3 (nm) | 51.41 ± 0.22 |
|  |  | Amplitude 3 (a.u.) | 6.34 ± 0.35 |
|  |  | Broadening (nm) | 7.65 ± 0.04 |
|  | **Repeated localisations** | Not included | |
