# Supplementary material for: Using iPALM to determine protein organisation in cardiac muscle Z-discs
Source: PLoS Comput Biol. 2026 Sep 28;22(9):e1014309. doi: 10.1371/journal.pcbi.1014309 (PMC13638043; doi:10.1371/journal.pcbi.1014309)
Supplement: Supplementary file 4 [file pcbi.1014309.s008.docx]

**S4 Table. Most likely models and parameter values for Z1Z2 arrangement.**

| **Axial** | **Background:** linear | Slope (a.u. nm^-1^) | 1.21 ± 0.19 |
| --- | --- | --- | --- |
|  |  | Offset (a.u.) | -0.01 ± 0.00 |
|  | **Characteristic distances:** linear repeat, 6 peaks | Repeat distance (nm) | 16.88 ± 0.05 |
|  |  | Amplitude 1 (a.u.) | 50.14 ± 4.08 |
|  |  | Amplitude 2 (a.u.) | 91.32 ± 4.23 |
|  |  | Amplitude 3 (a.u.) | 34.40 ± 3.12 |
|  |  | Amplitude 4 (a.u.) | 44.14 ± 2.85 |
|  |  | Amplitude 5 (a.u.) | 2.930 ± 1.63 |
|  |  | Amplitude 6 (a.u.) | 13.09 ± 1.30 |
|  |  | Broadening (nm) | 12.40 ± 0.37 |
|  | **Repeated localisations** | Amplitude (a.u.) | 59.97 ± 1.50 |
|  |  | Broadening (nm) | 7.85 ± 0.10 |
| **Transverse** | **Background:** linear_2D,int=0_ | Slope (a.u. nm^-1^) | 0.01 ± 0.00 |
|  | **Characteristic distances:** square distance ratios (a, √2a, a), 3 peaks | Unit distance (nm) | 19.14 ± 0.23 |
|  |  | Amplitude 1 (a.u.) | 3.73 ± 1.89 |
|  |  | Amplitude 2 (a.u.) | 1.38 ± 0.50 |
|  |  | Amplitude 3 (a.u.) | 7.36 ± 0.87 |
|  |  | Broadening (nm) | 7.37 ± 0.33 |
|  | **Repeated localisations** | Amplitude (a.u.) | 9.71 ± 3.08 |
|  |  | Broadening (nm) | 9.75 ± 1.38 |
