# Supplementary material for: Using iPALM to determine protein organisation in cardiac muscle Z-discs
Source: PLoS Comput Biol. 2026 Sep 28;22(9):e1014309. doi: 10.1371/journal.pcbi.1014309 (PMC13638043; doi:10.1371/journal.pcbi.1014309)
Supplement: Supplementary file 5 [file pcbi.1014309.s009.docx]

**Table S5.** **Most likely models and parameter values for ZASP arrangement.**

| **Axial** | **Background:** constant | Offset (a.u.) | 0.23 ± 0.0 |
| --- | --- | --- | --- |
|  | **Characteristic distances:** linear repeat, 5 peaks | Repeat distance (nm) | 18.22 ± 0.06 |
|  |  | Amplitude 1 (a.u.) | 15.06 ± 0.15 |
|  |  | Amplitude 2 (a.u.) | 4.64 ± 0.07 |
|  |  | Amplitude 3 (a.u.) | 2.7 ± 0.05 |
|  |  | Amplitude 4 (a.u.) | 2.03 ± 0.06 |
|  |  | Amplitude 5 (a.u.) | 2.93 ± 0.06 |
|  |  | Broadening (nm) | 9.68 ± 0.11 |
|  | **Repeated localisations** | Amplitude (a.u.) | 10.84 ± 0.12 |
|  |  | Broadening (nm) | 6.73 ± 0.06 |
| **Transverse** | **Background:** linear_2D,int=0_ | Slope (a.u. nm^-1^) | 0.01 ± 0.0 |
|  | **Characteristic distances:** linear repeat, 2 peaks | Unit distance (nm) | 28.17 ± 0.02 |
|  |  | Amplitude 1 (a.u.) | 5.11 ± 0.09 |
|  |  | Amplitude 2 (a.u.) | 1.32 ± 0.13 |
|  |  | Broadening (nm) | 8.96 ± 0.05 |
|  | **Repeated localisations** | Amplitude (a.u.) | 4.08 ± 0.01 |
|  |  | Broadening (nm) | 6.87 ± 0.0 |
