# Supplementary material for: Using iPALM to determine protein organisation in cardiac muscle Z-discs
Source: PLoS Comput Biol. 2026 Sep 28;22(9):e1014309. doi: 10.1371/journal.pcbi.1014309 (PMC13638043; doi:10.1371/journal.pcbi.1014309)
Supplement: Supplementary file 6 [file pcbi.1014309.s013.docx]

PCOMPBIOL-D-26-01083
Using iPALM to determine protein organisation in cardiac muscle Z-discs
PLOS Computational Biology


**Additional Editor Comments (if provided):**

**Journal Requirements:**

**Please provide an Author Summary**. **This should appear in your manuscript between the Abstract (if applicable) and the Introduction and should be 150-200 words long**. The aim should be to make your findings accessible to a wide audience that includes both scientists and non-scientists. Sample summaries can be found on our website under Submission Guidelines:

<https://journals.plos.org/ploscompbiol/s/submission-guidelines#loc-parts-of-a-submission>

This summary is now provided as requested.

3) We noticed that you used the **phrase 'data not shown'** in the manuscript. We do not allow these references, as the PLOS data access policy requires that all data be either published with the manuscript or made available in a publicly accessible database. Please amend the supplementary material to include the referenced data or remove the references.

As far as we are aware, all the data is shown in the manuscript. We think this comment might relate to wording in the legend for the model shown in Figure 3, which was worded clumsily– so we have changed this to make it clear.

4) We do not publish any copyright or **trademark symbols** that usually accompany proprietary names, eg ©,  ®, or TM  (e.g. next to drug or reagent names). Therefore please remove all instances of trademark/copyright symbols throughout the text, including:
**- TM on pages: 17, 18, and 19.**

These have been amended as required.

5) **Please upload all main figures as separate Figure files in .tif or .eps** format. For more information about how to convert and format your figure files please see our guidelines: 
<https://journals.plos.org/ploscompbiol/s/figures>

We have converted all our figures to .tif format (as per guidelines) and have uploaded.

6) Please upload a copy of **Figures FIG 1 A-C, 2A-F, and 3AB** which you refer to in your text on pages 6, 9, and 12. Or, if the figure is no longer to be included as part of the submission please remove all reference to it within the text.

These figures have been uploaded (as in 5).

7) We have noticed that you have uploaded Supporting Information files, but you have not included a **list of legends**. Please add a full list of legends for your Supporting Information files after the references list.

The legends were provided separately under each figure in Supporting information in our earlier version. We have now separated out each figure individually and uploaded, and we provide the legends for supporting figures and tables separately after the references.

8) We have amended your Competing Interest statement to comply with journal style. We kindly ask that you double check the statement and let us know if anything is incorrect.

**Reviewers' comments:**

Thank you to the reviewers for their comments.

Reviewer's Responses to Questions

**Comments to the Authors:
Please note here if the review is uploaded as an attachment.**

Reviewer #1: PCOMPBIOL-D-26-01083

I reviewed a manuscript by Oliver Umney/Alistair P. Curd et al., which uses adhirons against three Z-disc proteins and iPALM microscopy to understand better the molecular architecture of the cardiac Z-disc.
This is a well written and logical manuscript, combining adhirons against three Z-disc proteins. These are exciting new tools to improve resolution of light microscopy, in the current study applied to iPALM with PERPL. This work is of interest to a cardiac audience, but potentially also to wider audiences (in terms of the general technique).

Minor points that could be addressed to improve the study:

Introduction:
**The key super-resolution imaging techniques should be mentioned briefly (2nd paragraph page 4)**
**Some longer explanation what adhirons/affimers are (bottom page 4) would be beneficial. What makes them different to a nanobody?**

Additional text for both of these points has now been added to the relevant paragraphs in the introduction as requested.

Results:
**The cross-reactivity of the Actn2 adhiron to ZASP is non-neglectable** (Figure S1). In an ideal world, the adhirons would be validated on knock-out/knock-down cells or similar (C2C12?). At least this should be discussed.

We have now discussed this and provided a bit more detail about the mechanism of binding. The apparent cross-reactivity is likely due to the ‘stickiness’ of the Adhirons in pulldown assays, especially for loops that are largely hydrophobic. As the Adhirons recognise both sequence and native conformation, it is unlikely that they are really cross-reactive.

We performed additional SPR Assays in which we did not observe any binding of the actin Adhiron to the ZASP PDZ domains or Z1Z2 domains. The Adhirons are typically so highly specific and bind with nanomolar affinity, and we do not typically perform knockdown assays.

We have included two additional authors, who performed these experiments for us.

Page 6 middle “Interestingly, the density of localisations for ACTN2 was higher than those for Z1Z2 and lowest for ZASP.“ - **Doesn’t this also depend on the affinity of each adhiron to its target protein**? In this context, it might also be interesting to determine the affinity of the adhirons to their target and the cross-reactive proteins. How do these affinities compare to conventional antibodies?

The affinities of Adhirons for their targets is typically in the nanomolar range (see Tiede et al. references in the manuscript). We have now measured the apparent binding affinities of Z1Z2 and Zasp Adhirons to their targets by SPR (new Fig S1D), which show high nanomolar affinities (< 5nM). We now include example curves for these experiments in supplemental (Fig. S1D), and the measured apparent affinities for these interactions in the text. We did not perform this measurement for the ɑ-actinin-2 adhiron as we have already co-crystalised this with its target protein (published previously: PDB -6SWT), which also likely has nanomolar affinity.

Page 11 middle “**One possible explanation for this is that the PDZ domains of ZASP, […], separated by 16-18 nm … “ add a reference for this statement.**

A reference has been added (Ref 35).

Reviewer #2: This manuscript describes a pilot study to use Adhirons to delineate the organisation of the Z-disc in the striated muscle sarcomere. The Z-disc is known as a crucial structural and signalling hub and it contains hundreds of proteins. Currently there is extremely limited information on how they are assembled in 3D. Because it is an extremely crowded space, traditional labelling experiments using antibodies were not very successful because they are to big to penetrate much. Therefore the use of Adhirons is an extremely interesting experimental strategy. In this paper three adhirons were evaluated that recognise three different components of the Z-disc. Initial characterisation is described and then they are used on pig cardiac myofibrils and analysed using iPALM followed by PERPL (Pattern Extraction from Relative Positions of Localisations). Eventually a model is presented, where the three proteins are mapped onto a Z-disc.
While the data for the Adhirons against sarcomeric alpha-actinin and the titin Z1Z2 repeats look very good, **the ZASP Adhiron appears to be a much weaker binder**, so some of the proposed modelling may just be due to the reagent not working as well and there might be more ZASP around in reality. The n number for the ZASP is only 5 and the Adhiron seems to have a slight cross-reactivity with alpha-actinin (Supplementary Figure 1C), so the data with this reagent have to be looked at with a bit more caution.
It is extremely interesting that the authors conclude that the Z1Z2 repeat appears much more ordered than the alpha-actinin, since this may have implications for myofibrillogenesis.

While this could be the case, in fact the ZASP and Z1Z2 Adhirons both have low nanomolar affinity for their target proteins (as measured by SPR), making this unlikely. We now include this data in the revised paper (Fig. S1D: example curves, and apparent binding affinities in the text).

There are just two minor corrections that should be made:
1. **Throughout the manuscript the term “ACTN2” is used when they talk about sarcomeric alpha-actinin protein. ACTN2 is conventionally exclusively the name of the human gene and the protein is referred to either as sarcomeric alpha-actinin or alpha-actinin-2. Please change throughout the manuscript.**

This has been done.

2. Figure Legend S1: **There is no “C” described in the legend; also why is the MW of alpha-actinin 50 kDa and not 100 kDa?**

This is because we used a construct only containing the CH domains of alpha-actinin – (coupled to GST for the pulldown assays) and so the MW is correct for this construct. Apologies – but we think there was a legend for C. We have made sure this is there in the revised version of the paper.

**Have the authors made all data and (if applicable) computational code underlying the findings in their manuscript fully available?**
The [PLOS Data policy](https://eur03.safelinks.protection.outlook.com/?url=https%3A%2F%2Ftrack.editorialmanager.com%2FCL0%2Fhttps%3A%252F%252Fjournals.plos.org%252Fploscompbiol%252Fs%252Fmaterials-and-software-sharing%2F1%2F010f019f173629c1-37949b00-34a6-4c60-8ca0-6915c0276b89-000000%2FqN6SLDUmJU1_mR5u25GfnmJmQvvR_BbW-llTDdwXHuw%3D258&data=05%7C02%7Cm.peckham%40leeds.ac.uk%7C09b196cea99745e25fb908ded670bc91%7Cbdeaeda8c81d45ce863e5232a535b7cb%7C0%7C0%7C639183976865758632%7CUnknown%7CTWFpbGZsb3d8eyJFbXB0eU1hcGkiOnRydWUsIlYiOiIwLjAuMDAwMCIsIlAiOiJXaW4zMiIsIkFOIjoiTWFpbCIsIldUIjoyfQ%3D%3D%7C0%7C%7C%7C&sdata=uVhaRO%2FLuaLCOe7PwOb53ah1s%2FEpBCtwPSmEwCGSjOE%3D&reserved=0) requires authors to make all data and code underlying the findings described in their manuscript fully available without restriction, with rare exception (please refer to the Data Availability Statement in the manuscript PDF file). The data and code should be provided as part of the manuscript or its supporting information, or deposited to a public repository. For example, in addition to summary statistics, the data points behind means, medians and variance measures should be available. If there are restrictions on publicly sharing data or code —e.g. participant privacy or use of data from a third party—those must be specified.

Reviewer #1: Yes

Reviewer #2: Yes

**Figure resubmission:**

While revising your submission, we strongly recommend that you use PLOS’s NAAS tool (<https://ngplosjournals.pagemajik.ai/artanalysis>) to test your figure files. NAAS can convert your figure files to the TIFF file type and meet basic requirements (such as print size, resolution), or provide you with a report on issues that do not meet our requirements and that NAAS cannot fix.

Done
