# Supplementary material for: Using iPALM to determine protein organisation in cardiac muscle Z-discs
Source: PLoS Comput Biol. 2026 Sep 28;22(9):e1014309. doi: 10.1371/journal.pcbi.1014309 (PMC13638043; doi:10.1371/journal.pcbi.1014309)
Supplement: Peer review history

# Peer review: Decision Letter 0

**DOI:** 10.1371/journal.pcbi.1014309.r001

**Authors:** Boyce Griffith, Emma Lejeune

30 Jun 2026

PCOMPBIOL-D-26-01083

Using iPALM to determine protein organisation in cardiac muscle Z-discs

PLOS Computational Biology

Dear Dr. Peckham,

Thank you for submitting your manuscript to PLOS Computational Biology. After careful consideration, we feel that it has merit but does not fully meet PLOS Computational Biology's publication criteria as it currently stands. Therefore, we invite you to submit a revised version of the manuscript that addresses the points raised during the review process.

Please submit your revised manuscript by Jul 30 2026 11:59PM. If you will need more time than this to complete your revisions, please reply to this message or contact the journal office at ploscompbiol@plos.org. When you're ready to submit your revision, log on to https://www.editorialmanager.com/pcompbiol/ and select the 'Submissions Needing Revision' folder to locate your manuscript file.

Please include the following items when submitting your revised manuscript:

* A letter that responds to each point raised by the editor and reviewer(s). You should upload this letter as a separate file labeled 'Response to Reviewers'. This file does not need to include responses to formatting updates and technical items listed in the 'Journal Requirements' section below.

* A marked-up copy of your manuscript that highlights changes made to the original version. You should upload this as a separate file labeled 'Revised Manuscript with Track Changes'.

* An unmarked version of your revised paper without tracked changes. You should upload this as a separate file labeled 'Manuscript'.

If you would like to make changes to your financial disclosure, competing interests statement, or data availability statement, please make these updates within the submission form at the time of resubmission. Guidelines for resubmitting your figure files are available below the reviewer comments at the end of this letter.

As the corresponding author, your ORCID iD is verified in the submission system and will appear in the published article. PLOS supports the use of ORCID, and we encourage all coauthors to register for an ORCID iD and use it as well. Please encourage your coauthors to verify their ORCID iD within the submission system before final acceptance, as unverified ORCID iDs will not appear in the published article. Only the individual author can complete the verification step; PLOS staff cannot verify ORCID iDs on behalf of authors.

We look forward to receiving your revised manuscript.

Kind regards,

Emma Lejeune

Academic Editor

PLOS Computational Biology

Boyce Griffith

Section Editor

PLOS Computational Biology

**Additional Editor Comments (if provided):**

**Journal Requirements:**

If the reviewer comments include a recommendation to cite specific previously published works, please review and evaluate these publications to determine whether they are relevant and should be cited. There is no requirement to cite these works unless the editor has indicated otherwise.

1) We ask that a manuscript source file is provided at Revision. Please upload your manuscript file as a .doc, .docx, .rtf or .tex. If you are providing a .tex file, please upload it under the item type u2018LaTeX Source Fileu2019 and leave your .pdf version as the item type u2018Manuscriptu2019.

2) Please provide an Author Summary. This should appear in your manuscript between the Abstract (if applicable) and the Introduction, and should be 150-200 words long. The aim should be to make your findings accessible to a wide audience that includes both scientists and non-scientists. Sample summaries can be found on our website under Submission Guidelines:

https://journals.plos.org/ploscompbiol/s/submission-guidelines#loc-parts-of-a-submission

3) We noticed that you used the phrase 'data not shown' in the manuscript. We do not allow these references, as the PLOS data access policy requires that all data be either published with the manuscript or made available in a publicly accessible database. Please amend the supplementary material to include the referenced data or remove the references.

4) We do not publish any copyright or trademark symbols that usually accompany proprietary names, eg ©,  ®, or TM  (e.g. next to drug or reagent names). Therefore please remove all instances of trademark/copyright symbols throughout the text, including:

- TM on pages: 17, 18, and 19.

5) Please upload all main figures as separate Figure files in .tif or .eps format. For more information about how to convert and format your figure files please see our guidelines:

https://journals.plos.org/ploscompbiol/s/figures

6) Please upload a copy of Figures FIG 1 A-C, 2A-F, and 3AB which you refer to in your text on pages 6, 9, and 12. Or, if the figure is no longer to be included as part of the submission please remove all reference to it within the text.

7) We have noticed that you have uploaded Supporting Information files, but you have not included a list of legends. Please add a full list of legends for your Supporting Information files after the references list.

8)We have amended your Competing Interest statement to comply with journal style. We kindly ask that you double check the statement and let us know if anything is incorrect.

**Reviewers' comments:**

Reviewer's Responses to Questions

**Comments to the Authors:**

**Please note here if the review is uploaded as an attachment.**

Reviewer #1: PCOMPBIOL-D-26-01083

I reviewed a manuscript by Oliver Umney/Alistair P. Curd et al., which uses adhirons against three Z-disc proteins and iPALM microscopy to understand better the molecular architecture of the cardiac Z-disc.

This is a well written and logical manuscript, combining adhirons against three Z-disc proteins. These are exciting new tools to improve resolution of light microscopy, in the current study applied to iPALM with PERPL. This work is of interest to a cardiac audience, but potentially also to wider audiences (in terms of the general technique).

Minor points that could be addressed to improve the study:

Introduction:

The key super-resolution imaging techniques should be mentioned briefly (2nd paragraph page 4)

Some longer explanation what adhirons/affimers are (bottom page 4) would be beneficial. What makes them different to a nanobody?

Results:

The cross-reactivity of the Actn2 adhiron to ZASP is non-neglectable (Figure S1). In an ideal world, the adhirons would be validated on knock-out/knock-down cells or similar (C2C12?). At least this should be discussed.

Page 6 middle “Interestingly, the density of localisations for ACTN2 was higher than those for Z1Z2 and lowest for ZASP.“ - Doesn’t this also depend on the affinity of each adhiron to its target protein? In this context, it might also be interesting to determine the affinity of the adhirons to their target and the cross-reactive proteins. How do these affinities compare to conventional antibodies?

Page 11 middle “One possible explanation for this is that the PDZ domains of ZASP, […], separated by 16-18 nm … “ add a reference for this statement.

Reviewer #2: This manuscript describes a pilot study to use Adhirons to delineate the organisation of the Z-disc in the striated muscle sarcomere. The Z-disc is known as a crucial structural and signalling hub and it contains hundreds of proteins. Currently there is extremely limited information on how they are assembled in 3D. Because it is an extremely crowded space, traditional labelling experiments using antibodies were not very successful because they are to big to penetrate much. Therefore the use of Adhirons is an extremely interesting experimental strategy. In this paper three adhirons were evaluated that recognise three different components of the Z-disc. Initial characterisation is described and then they are used on pig cardiac myofibrils and analysed using iPALM followed by PERPL (Pattern Extraction from Relative Positions of Localisations). Eventually a model is presented, where the three proteins are mapped onto a Z-disc.

While the data for the Adhirons against sarcomeric alpha-actinin and the titin Z1Z2 repeats look very good, the ZASP Adhiron appears to be a much weaker binder, so some of the proposed modelling may just be due to the reagent not working as well and there might be more ZASP around in reality. The n number for the ZASP is only 5 and the Adhiron seems to have a slight cross-reactivity with alpha-actinin (Supplementary Figure 1C), so the data with this reagent have to be looked at with a bit more caution.

It is extremely interesting that the authors conclude that the Z1Z2 repeat appears much more ordered than the alpha-actinin, since this may have implications for myofibrillogenesis.

There are just two minor corrections that should be made:

1. Throughout the manuscript the term “ACTN2” is used when they talk about sarcomeric alpha-actinin protein. ACTN2 is conventionally exclusively the name of the human gene and the protein is referred to either as sarcomeric alpha-actinin or alpha-actinin-2. Please change throughout the manuscript.

2. Figure Legend S1: There is no “C” described in the legend; also why is the MW of alpha-actinin 50 kDa and not 100 kDa?

**********

**Have the authors made all data and (if applicable) computational code underlying the findings in their manuscript fully available?**

The PLOS Data policy requires authors to make all data and code underlying the findings described in their manuscript fully available without restriction, with rare exception (please refer to the Data Availability Statement in the manuscript PDF file). The data and code should be provided as part of the manuscript or its supporting information, or deposited to a public repository. For example, in addition to summary statistics, the data points behind means, medians and variance measures should be available. If there are restrictions on publicly sharing data or code —e.g. participant privacy or use of data from a third party—those must be specified.

Reviewer #1: Yes

Reviewer #2: Yes

**********

PLOS authors have the option to publish the peer review history of their article (what does this mean?). If published, this will include your full peer review and any attached files.

If you choose “no”, your identity will remain anonymous but your review may still be made public.

**Do you want your identity to be public for this peer review?**  For information about this choice, including consent withdrawal, please see our Privacy Policy.

Reviewer #1: No

Reviewer #2: No

[NOTE: If reviewer comments were submitted as an attachment file, they will be attached to this email and accessible via the submission site. Please log into your account, locate the manuscript record, and check for the action link "View Attachments". If this link does not appear, there are no attachment files.]

**Figure resubmission:**

While revising your submission, we strongly recommend that you use PLOS’s NAAS tool (https://ngplosjournals.pagemajik.ai/artanalysis) to test your figure files. NAAS can convert your figure files to the TIFF file type and meet basic requirements (such as print size, resolution), or provide you with a report on issues that do not meet our requirements and that NAAS cannot fix.

After uploading your figures to PLOS’s NAAS tool - https://ngplosjournals.pagemajik.ai/artanalysis, NAAS will process the files provided and display the results in the "Uploaded Files" section of the page as the processing is complete. If the uploaded figures meet our requirements (or NAAS is able to fix the files to meet our requirements), the figure will be marked as "fixed" above. If NAAS is unable to fix the files, a red "failed" label will appear above. When NAAS has confirmed that the figure files meet our requirements, please download the file via the download option, and include these NAAS processed figure files when submitting your revised manuscript.

**Reproducibility:**

To enhance the reproducibility of your results, we recommend that authors of applicable studies deposit laboratory protocols in protocols.io, where a protocol can be assigned its own identifier (DOI) such that it can be cited independently in the future. Additionally, PLOS ONE offers an option to publish peer-reviewed clinical study protocols. Read more information on sharing protocols at https://plos.org/protocols?utm_medium=editorial-email&utm_source=authorletters&utm_campaign=protocols

# Author comment: Author response to Decision Letter 1

**DOI:** 10.1371/journal.pcbi.1014309.r002

17 Aug 2026

Attachment Submitted filename: PCOMPBIOL_response..docx

# Peer review: Decision Letter 1

**DOI:** 10.1371/journal.pcbi.1014309.r003

**Authors:** Boyce Griffith, Emma Lejeune

17 Sep 2026

Dear Dr. Peckham,

We are pleased to inform you that your manuscript 'Using iPALM to determine protein organisation in cardiac muscle Z-discs' has been provisionally accepted for publication in PLOS Computational Biology.

Before your manuscript can be formally accepted you will need to complete some formatting changes, which you will receive in a follow up email. A member of our team will be in touch with a set of requests.

Please note that your manuscript will not be scheduled for publication until you have made the required changes, so a swift response is appreciated.

IMPORTANT: The editorial review process is now complete. PLOS will only permit corrections to spelling, formatting or significant scientific errors from this point onwards. Requests for major changes, or any which affect the scientific understanding of your work, will cause delays to the publication date of your manuscript.

Should you, your institution's press office or the journal office choose to press release your paper, you will automatically be opted out of early publication. We ask that you notify us now if you or your institution is planning to press release the article. All press must be co-ordinated with PLOS.

Thank you again for supporting Open Access publishing; we are looking forward to publishing your work in PLOS Computational Biology.

Best regards,

Emma Lejeune

Academic Editor

PLOS Computational Biology

Boyce Griffith

Section Editor

PLOS Computational Biology

***********************************************************

Reviewer's Responses to Questions

**Comments to the Authors:**

**Please note here if the review is uploaded as an attachment.**

Reviewer #1: The revision has improved the manuscript and addressed my points raised. Rebuttal very clear.

Congratulations to the authors!

Reviewer #2: My comments were all addressed in the revised version of the manuscript.

**********

**Have the authors made all data and (if applicable) computational code underlying the findings in their manuscript fully available?**

The PLOS Data policy requires authors to make all data and code underlying the findings described in their manuscript fully available without restriction, with rare exception (please refer to the Data Availability Statement in the manuscript PDF file). The data and code should be provided as part of the manuscript or its supporting information, or deposited to a public repository. For example, in addition to summary statistics, the data points behind means, medians and variance measures should be available. If there are restrictions on publicly sharing data or code —e.g. participant privacy or use of data from a third party—those must be specified.

Reviewer #1: Yes

Reviewer #2: Yes

**********

PLOS authors have the option to publish the peer review history of their article (what does this mean?). If published, this will include your full peer review and any attached files.

If you choose “no”, your identity will remain anonymous but your review may still be made public.

**Do you want your identity to be public for this peer review?**  For information about this choice, including consent withdrawal, please see our Privacy Policy.

Reviewer #1: No

Reviewer #2: No

# Editor report: Acceptance letter

**DOI:** 10.1371/journal.pcbi.1014309.r004

**Authors:** Boyce Griffith, Emma Lejeune

PCOMPBIOL-D-26-01083R1

Using iPALM to determine protein organisation in cardiac muscle Z-discs

Dear Dr Peckham,

I am pleased to inform you that your manuscript has been formally accepted for publication in PLOS Computational Biology. Your manuscript is now with our production department and you will be notified of the publication date in due course.

The corresponding author will soon be receiving a typeset proof for review, to ensure errors have not been introduced during production. Please review the PDF proof of your manuscript carefully, as this is the last chance to correct any errors. Please note that major changes, or those which affect the scientific understanding of the work, will likely cause delays to the publication date of your manuscript.

Soon after your final files are uploaded, unless you have opted out, the early version of your manuscript will be published online. The date of the early version will be your article's publication date. The final article will be published to the same URL, and all versions of the paper will be accessible to readers.

For Research, Software, and Methods articles, you will receive an invoice from PLOS for your publication fee after your manuscript has reached the completed accept phase. If you receive an email requesting payment before acceptance or for any other service, this may be a phishing scheme. Learn how to identify phishing emails and protect your accounts at https://explore.plos.org/phishing.

Thank you again for supporting PLOS Computational Biology and open-access publishing. We are looking forward to publishing your work!

With kind regards,

Sharmila Kamatchi

PLOS Computational Biology | Carlyle House, Carlyle Road, Cambridge CB4 3DN | United Kingdom ploscompbiol@plos.org | Phone +44 (0) 1223-442824 | ploscompbiol.org | @PLOSCompBiol
